# Evaluation of emotional arousal level and depression severity using voice-derived sound pressure change acceleration

**DOI:** 10.1038/s41598-021-92982-7

**Published:** 2021-06-30

**Authors:** Shuji Shinohara, Hiroyuki Toda, Mitsuteru Nakamura, Yasuhiro Omiya, Masakazu Higuchi, Takeshi Takano, Taku Saito, Masaaki Tanichi, Shuken Boku, Shunji Mitsuyoshi, Mirai So, Aihide Yoshino, Shinichi Tokuno

**Affiliations:** 1 Department of Bioengineering, Graduate School of Engineering, The University of Tokyo, 7-3-1 Hongo, Bunkyo-ku, Tokyo 113-8656 Japan; 2 Department of Psychiatry, National Defense Medical College, 3-2 Namiki, Tokorozawa, Saitama 359-8513 Japan; 3 PST Inc., Industry & Trade Center Building 905, 2 Yamashita-cho, Naka-ku, Yokohama, Kanagawa 231-0023 Japan; 4 Department of Neuropsychiatry, Faculty of Life Sciences, Kumamoto University, 1-1-1 Honjo, Chuo-ku, Kumamoto, Kumamoto 860-8556 Japan; 5 Department of Psychiatry, Tokyo Dental College, 2-9-18, Misakicho, Chiyoda-ku, Tokyo 101-0061 Japan

**Keywords:** Psychology, Biomarkers, Health care, Disease prevention

## Abstract

In this research, we propose a new index of emotional arousal level using sound pressure change acceleration, called the emotional arousal level voice index (EALVI), and investigate the relationship between this index and depression severity. First, EALVI values were calculated from various speech recordings in the interactive emotional dyadic motion capture database, and the correlation with the emotional arousal level of each voice was examined. The resulting correlation coefficient was 0.52 (n = 10,039, *p* < 2.2** × **10^−16^). We collected a total of 178 datasets comprising 10 speech phrases and the Hamilton Rating Scale for Depression (HAM-D) score of outpatients with major depression at the Ginza Taimei Clinic (GTC) and the National Defense Medical College (NDMC) Hospital. The correlation coefficients between the EALVI and HAM-D scores were − 0.33 (n = 88, *p* = 1.8** × **10^−3^) and − 0.43 (n = 90, *p* = 2.2** × **10^−5^) at the GTC and NDMC, respectively. Next, the dataset was divided into “no depression” (HAM-D < 8) and “depression” groups (HAM-D ≥ 8) according to the HAM-D score. The number of patients in the “no depression” and “depression” groups were 10 and 78 in the GTC data, and 65 and 25 in the NDMC data, respectively. There was a significant difference in the mean EALVI values between the two groups in both the GTC and NDMC data (*p* = 8.9** × **10^−3^, Cliff’s delta = 0.51 and *p* = 1.6** × **10^−3^; Cliff’s delta = 0.43, respectively). The area under the curve of the receiver operating characteristic curve when discriminating both groups by EALVI was 0.76 in GTC data and 0.72 in NDMC data. Indirectly, the data suggest that there is some relationship between emotional arousal level and depression severity.

## Introduction

The relationship between depression and emotional arousal has been established through studies using neurophysiological tests such as electroencephalogram (e.g., late positive potential amplitude), magnetoencephalography (e.g., neuromagnetic oscillatory activity), and skin conductance response^1–3^. Consequently, with the explosive increase in smartphone usage, research on voice emotion recognition and measurement of emotional arousal level using voice have been encouraged. For example, the relationship between emotional arousal level and voice intensity or pitch has been documented^4,5^. On the other hand, the voice of a depressed person has dull, monotonous, and lifeless features^6^, and listeners can perceive patients’ distinctive prosody^7,8^. If we can measure emotional arousal level by voice and determine the presence or absence of depression, we may be able to detect depression remotely using a smartphone. Additionally, if it becomes possible to objectively assess the presence or absence of depression using voice in future, it may be possible to detect unconscious pre-depressed people at a very early stage.

The Hamilton Rating Scale for Depression (HAM-D)^9^ and self-administered questionnaires such as the General Health Questionnaire^10^ and the Beck Depression Inventory^11^ are powerful tools for measuring depression. However, voice monitoring has the advantage of being more sensitive to daily changes than self-administered questionnaires.

From this perspective, several studies have been conducted to measure depression severity using voice^12^; these have shown that speech characteristics are effective predictors of the signs and severity of depression^13^.

Similarly, Cannizzaro et al.^14^ examined the relationship between the HAM-D score and voice, and found a strong correlation between the HAM-D score and speaking rate or pitch variation. Yang et al.^8^ demonstrated that changes in depression severity measured by the HAM-D, can be captured by the switching pause, that is, the pause duration between the end of one speaker’s utterance and the start of an utterance by the other. The mel-frequency cepstral coefficient (MFCC) is often used for voice recognition. Taguchi et al.^15^ showed that MFCC2 (the second dimension of MFCC) is effective in classifying patients with depression and individuals without depression.

In a previous study, we found that the higher the emotional arousal level, the higher were both the Hurst exponent and the zero-crossing rate of the waveform^16^. Specifically, we have shown that emotional arousal level can be approximated by a weighted average of the Hurst exponent and zero-crossing rate. The zero-crossing rate is the rate at which the signal crosses the reference line, and is small in a smooth curve such as a sine curve and large in a rough waveform such as white noise. However, the Hurst exponent is expressed as 2-D, where D is the fractal dimension that represents the complexity of the waveform. In other words, the Hurst exponent is a measure of smoothness, which is the opposite of the fractal dimension, and theoretically it is 0 for white noise and 0.5 for brown noise.

The purpose of this study is to propose a new voice index of emotional arousal using the acceleration of sound pressure level change and to investigate its potential for measuring depression severity. First, we propose a new speech feature called the emotional arousal level voice index (EALVI) that combines both the roughness and smoothness of the waveform using sound pressure change acceleration. Next, the EALVI was calculated from the emotional speech recordings stored in the interactive emotional dyadic motion capture (IEMOCAP) database^17^, and compared with the emotional arousal level evaluated by the annotators. Self-assessment manikins were used to evaluate the emotional arousal level (1-calm, 5-excited) in IEMOCAP^18^. This scheme consists of five figures that describe progressive changes in the attribute axis. The evaluators were asked to select the manikin that better describes the stimulus, which is mapped into an integer between 1 and 5 (from left to right). Next, the EALVI was calculated from the voice of the depressed patient and compared with the HAM-D score. The dataset used in this study is the same as the dataset used in Shinohara et al.^16^. We compare the performance of the EALVI with the arousal level voice index (ALVI) proposed in Shinohara et al.^16^ in the discussion section.

## Methods and materials

### Acquisition of data

#### Data on emotional arousal level

We used the IEMOCAP database^17^ to investigate the relationship between the proposed voice index, the EALVI, and emotional arousal. The database contains audio recordings of dyadic mixed-gender pairs of voice over artists. This database contains voices for five sessions in total, that is, five male and five female voices. Two different approaches were taken to ensure that genuine emotions were expressed in the dialogue: the use of plays (scripted sessions), and improvisation-based hypothetical scenarios (spontaneous sessions). The participants were seven professional actors and three senior students from the Drama Department at the University of Southern California (USC), and were selected after reviewing their audition sessions. The evaluators were USC students who were fluent English speakers.

The voices were manually divided into utterances (i.e., the sounds/words made from one breath to the next). There were 10,039 utterances in total. The emotional arousal level of each utterance was evaluated by at least two different annotators on a five-point scale. The emotional arousal level of each utterance was calculated as the average of the evaluation values given by each annotator. The Cronbach’s alpha coefficients^19^ were computed to test the reliabilities of the evaluations between the raters. The coefficient was 0.607.

Furthermore, emotion categories were evaluated by at least three annotators. There were nine emotion categories: “angry,” “happy,” “sad,” “neutral,” “frustrated,” “excited,” “fearful,” “surprised,” and “disgusted.” Too many categories of emotions would result in low agreement among raters. On the other hand, if the number is too small, the emotions cannot be expressed accurately. To balance this tradeoff, the final emotional categories selected for annotation were anger, sadness, happiness, disgust, fear, and surprise, which are known as basic emotions^20^, plus frustration, excited, and neutral states.

However, utterances that did not seem to fit into any of these categories were classified as “other.” A simple majority voting method was used to assign an emotion category if there was disagreement among annotators regarding classification. The annotators were also allowed to tag more than one emotion category. If no majority category could be assigned, the category was labeled xxx. The total number of utterances assigned to the nine emotion categories by the above procedure was 7527. To analyze the inter-evaluator agreement, Fleiss’ Kappa statistic^21^ was computed. The result for the entire database is κ = 0.27.

#### Data on severity of depression

This study collected data from outpatients with major depressive disorder (MDD) after obtaining written informed consent from all participants at the Ginza Taimei Clinic (GTC) and the National Defense Medical College (NDMC) Hospital. At each health care facility, the recruited patients were instructed to pronounce 17 Japanese phrases. However, the 17 phrases collected at the two hospitals were not exactly the same. Of the 17 phrases, 10 were common. For the purpose of this study, these 10 phrases were used for analysis. Table 1 shows the contents of these 10 phrases.

**Table 1:** Ten phrases used for analysis.

Phrase number	Phrase in Japanese	Purpose (meaning)
P1	I-ro-ha-ni-ho-he-to	Non-emotional (similar to “a-b-c”)
P2	Honjitsu ha seiten nari	Non-emotional (It is fine today)
P5	Mukashi aru tokoro ni	Non-emotional (Once upon a time, there lived)
P11	Garapagosu shotou	Check pronunciation (Galápagos Islands)
P12	Tsukarete guttari shiteimasu	Emotional (I am tired/dead tired)
P13	Totemo genki desu	Emotional (I am very cheerful)
P14	Kinou ha yoku nemuremashita	Emotional (I was able to sleep well yesterday)
P15	Shokuyoku ga arimasu	Emotional (I have an appetite)
P16	Okorippoi desu	Emotional (I am irritable)
P17	Kokoroga odayaka desu	Emotional (My heart is calm)

Voice was recorded using a pin microphone (ME52W, Olympus, Tokyo, Japan) attached to the patient’s chest, approximately 15 cm from the mouth. The recording equipment was a portable recorder R-26 (Roland, Shizuoka, Japan). The record format involved a linear pulse-code modulation (PCM). The sampling frequency and number of quantization bits were 11,025 Hz and 16, respectively.

We also had a doctor interview each patient and provide a score on the HAM-D, in the same session the voice recordings were used, to evaluate the severity of depression in patients. In this way, pairs of recorded voices of 10 phrases and HAM-D scores were collected from 178 patients. Table 2 shows the participants’ information from each health care facility. Patients were included if they had been diagnosed with MDD according to the Diagnostic and Statistical Manual of Mental Disorders, Fourth Edition, Text Revision^22^ and were aged over 20 years. They were excluded if they had been diagnosed with serious physical disorders or organic brain disease. They were diagnosed by a psychiatrist using the Mini-International Neuropsychiatric Interview^23^.

**Table 2:** Participants’ information.

Health care facility	Sex	Number of participants		Mean age ± SD	
GTC	Female	55	88	31.6 ± 8.6	32.0 ± 7.9
	Male	33		32.5 ± 6.5	
NDMC	Female	44	90	62.0 ± 13.1	55.2 ± 14.8
	Male	46		48.8 ± 13.5	

The protocol of this study was designed in accordance with the Declaration of Helsinki and relevant domestic guidelines issued by the concerned authorities, in Japan. The protocol was approved by the ethics committee of the Faculty of Medicine, the University of Tokyo (no. 11572), and the ethics committee of the National Defense Medical College (no. 2248).

### Proposed method

Consider a signal of sound pressure level $x\left( t \right),\;0 \le t \le T$. The velocity of change in sound pressure $v\left( t \right)$ at time “t” is defined as follows:

1 $$v\left( t \right) = \frac{{x\left( t \right) - x\left( {t - 1} \right)}}{{\Delta t}}$$

Next, the acceleration of sound pressure change $a\left( t \right)$ is defined as follows:

2 $$a\left( t \right) = \frac{1}{{mean\left( {\left| {v\left( {t + 1} \right)} \right|,\;\left| {v\left( t \right)} \right|} \right)}}\frac{{v\left( {t + 1} \right) - v\left( t \right)}}{{\Delta t}}$$

where $mean\left( {x,\;y} \right)$ represents the mean of x and y and is used for normalization. In this study, the harmonic mean is used as the mean. For simplicity, we set $\Delta t = 1$.

At this time, Eq. (2) can be rewritten as follows:

3 $$a\left( t \right) = \frac{{\left| {v\left( {t + 1} \right)} \right| + \left| {v\left( t \right)} \right|}}{{2\left| {v\left( {t + 1} \right)} \right|\left| {v\left( t \right)} \right|}}\left[ {v\left( {t + 1} \right) - v\left( t \right)} \right]$$

Next, we focus on the directionality of $a\left( t \right)$. The center line is defined as the average time $\bar{x}$ of the signal as follows:

4 $$\bar{x} = \frac{{\sum\nolimits_{{t = 0}}^{T} {x\left( t \right)} }}{{T + 1}}$$

**Figure 1:**
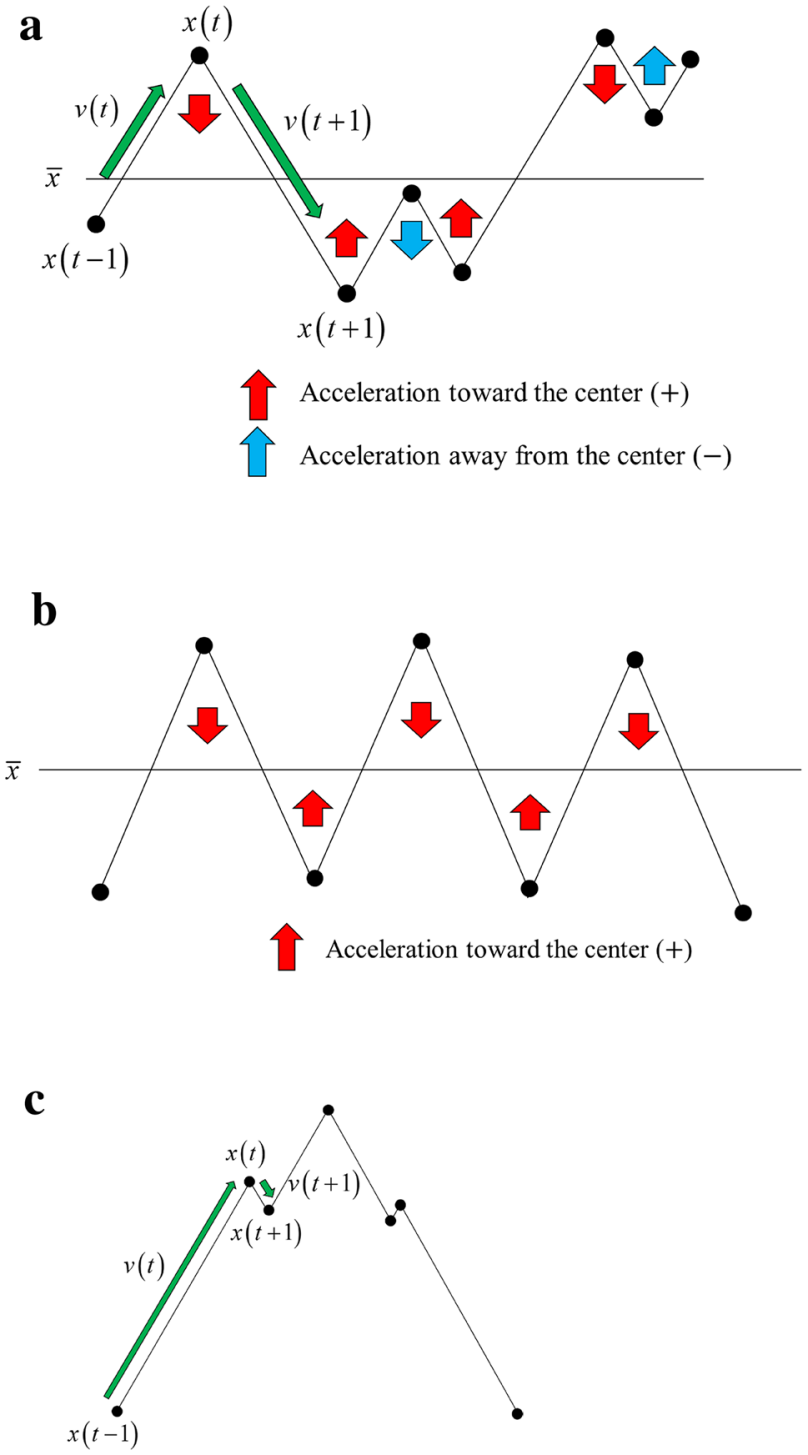
(**a**) Direction of acceleration. *Note*: Acceleration toward the center is positive, and acceleration away from the center is negative. (**b**) Acceleration for rough waveform. *Note*: The more the waveform crosses the centerline, the greater the proportion of acceleration towards the center. (**c**) Waveform when there is a bias between two adjacent velocity vectors. *Note*: When the absolute values $\left| {v\left( t \right)} \right|$ and $\left| {v\left( {t + 1} \right)} \right|$ of two adjacent velocity vectors differ greatly, the entire waveform tends to be smooth.

In the case of $a\left( t \right) < 0$, $a\left( t \right)$ represents the downward acceleration. Therefore, when $x\left( t \right)$ is above the center line, i.e., when $x\left( t \right) - \bar{x} > 0$, $a\left( t \right)$ represents the acceleration toward the center. Correspondingly, in the case of $x\left( t \right) - \bar{x} < 0$, $a\left( t \right)$ becomes an acceleration away from the center. In addition, in the case of $a\left( t \right) > 0$, $a\left( t \right)$ represents an upward acceleration; when $x\left( t \right) - \bar{x} < 0$, $a\left( t \right)$ represents the acceleration toward the center. Accordingly, when $x\left( t \right) - \bar{x} > 0$, $a\left( t \right)$ represents the acceleration away from the center. The EALVI value represents the acceleration, which is positive when toward the center and negative when away from the center (Fig. 1a). It is defined as follows:

5 $$EALVI\left( t \right) = \left\{ {\begin{array}{*{20}l} { - a\left( t \right)} \hfill & {if\;x\left( t \right) - \bar{x} > 0} \hfill \\ {a\left( t \right)} \hfill & {otherwise} \hfill \\ \end{array} } \right.$$

The EALVI value of a given voice signal is defined as the average time of $EALVI\left( t \right)$ as follows:

6 $$EALVI = \frac{{\sum\nolimits_{{t = 1}}^{{T - 1}} {EALVI\left( t \right)} }}{{T - 1}}$$

As is clear from the definition, the EALVI value increases as the proportion of acceleration toward the center increases. In addition, as can be seen by comparing Fig. 1a,b, the higher the ratio of the waveform crossing the center line, that is, the higher the zero crossing ratio, the larger the ratio of the acceleration toward the center. Thus, the EALVI can be said to be a measure that reflects the roughness of the waveform, like the zero-crossing rate.

Next, we focus on the first term $\frac{{\left| {v\left( {t + 1} \right)} \right| + \left| {v\left( t \right)} \right|}}{{2\left| {v\left( {t + 1} \right)} \right|\left| {v\left( t \right)} \right|}}$ on the right side of the Eq. (3). This term is the reciprocal of the harmonic mean of $\left| {v\left( t \right)} \right|$ and $\left| {v\left( {t + 1} \right)} \right|$. The harmonic mean of x and y is expressed as $2xy/\left( {x + y} \right)$, and the more skewed the values of x and y, the smaller the harmonic mean. Conversely, the more biased the values of $\left| {v\left( t \right)} \right|$ and $\left| {v\left( {t + 1} \right)} \right|$, the greater the EALVI value, and this increases as the bias between them increases. As can be seen by comparing Fig. 1b,c, the larger the deviation between the values of $\left| {v\left( t \right)} \right|$ and $\left| {v\left( {t + 1} \right)} \right|$, the smoother the entire waveform tends to be. In other words, the first term on the right side of Eq. (3) can be considered as a measure of smoothness. Thus, the EALVI can be regarded as a measure that reflects roughness and smoothness.

Because of the normalization process in Eq. (3), the EALVI is invariant with respect to scaling in the amplitude direction. For example, if C is a constant and the signal oscillates between − C and C, then EALVI = 2, regardless of the value of C. Furthermore, the EALVI tends to be larger for white noise and smaller for smooth waveforms such as Brown noise.

### Statistical analyses

Wilcoxon’s rank sum test was used to test the difference in means. Cliff’s delta was used as the effect size. The area under the curve (AUC) of the receiver operating characteristic curve was used to evaluate discrimination performance of the EALVI. For all analyses statistical significance was set at *p* < 0.05.

The following analysis was performed using the statistical software R, version 3.6.1 (2019-07-05)^24^, unless otherwise specified. We used the R packages of Epi version 2.41 for AUC calculation, exactRankTests version 0.8.31 for the Wilcoxon rank sum test, effsize version 0.8.1 for Cliff’s delta, and car version 3.0.8 for multicollinearity in multiple regression analysis.

The operating system used was Windows 10.

## Results

### EALVI as an emotional arousal level index

EALVI values were calculated from 10,039 utterances stored in the IEMOCAP database. The correlation coefficient between the EALVI and emotional arousal level of each utterance given by the annotators was 0.52 (n = 10,039, *p* < 2.2** × **10^−16^). We extracted low emotional arousal voices with a level of 2 or less (n = 1112, mean ± SD = 1.92 ± 0.19) and high emotional arousal voices with a level of 4 or more (n = 1692, mean ± SD = 4.19 ± 0.28), from the database. As a result of distinguishing these two groups by the EALVI, the AUC was 0.93 and when the cutoff value was 0.90, both the sensitivity and specificity were 0.86.

**Figure 2:**
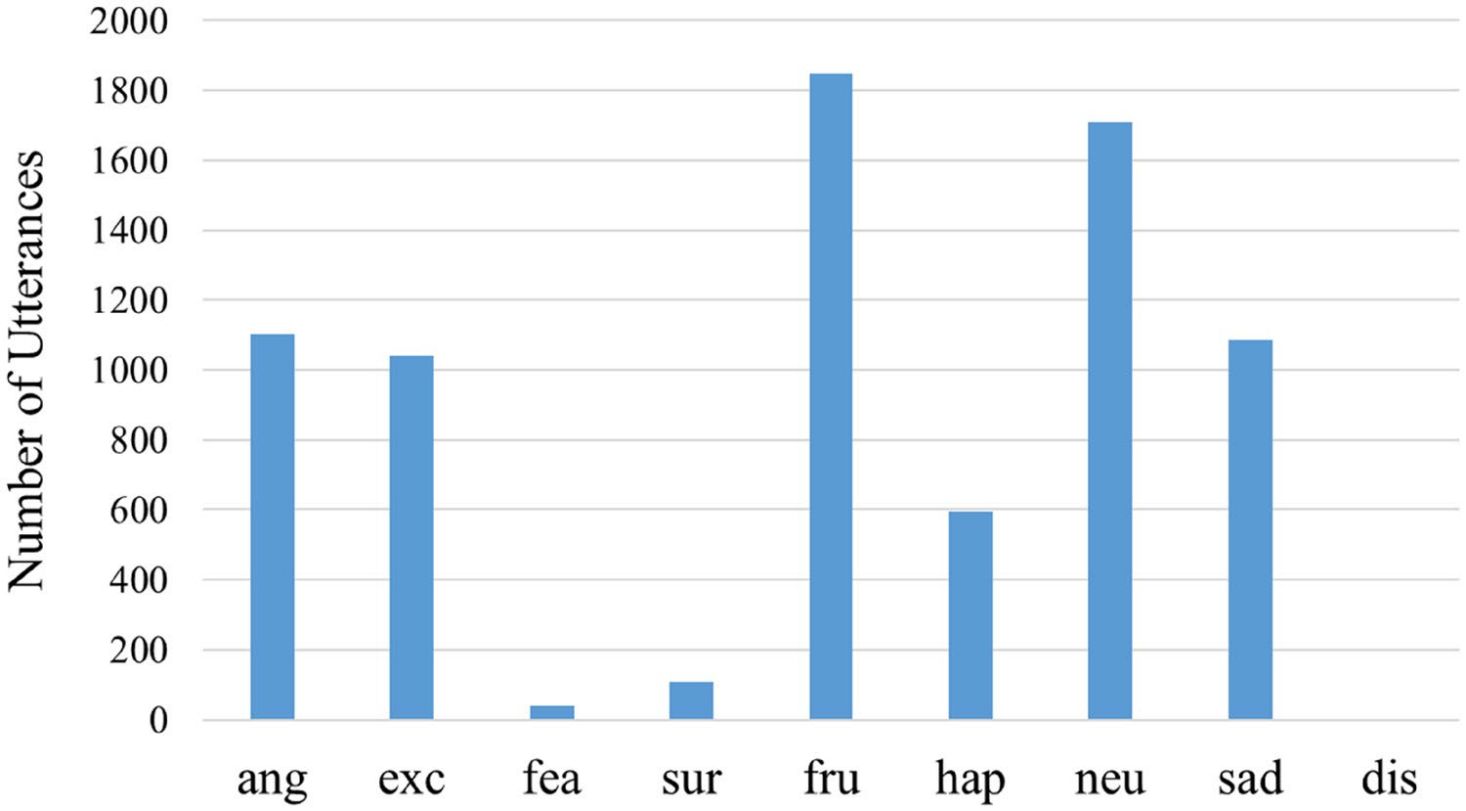
The number of utterances for each emotion category. *Note*: The number of utterances classified in “disgusted” was 2. Additionally, ang = angry; exc = excited; fea = fearful; sur = surprised; fru = frustrated; hap = happy; neu = neutral; sad = sad; dis = disgusted.

**Figure 3:**
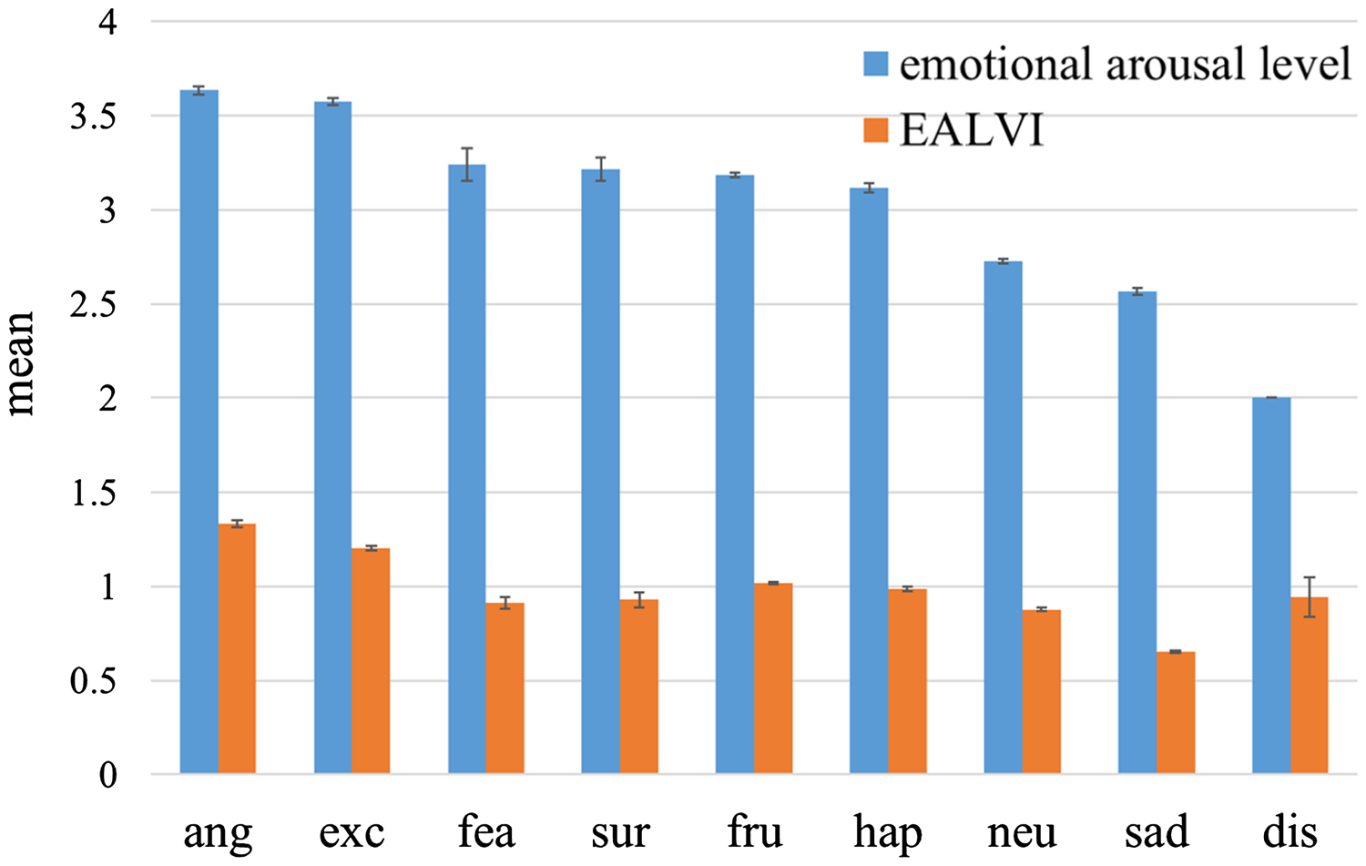
The mean emotional arousal level and mean EALVI value for each emotion category. *Note*: Error bars represent standard error. Additionally, ang = angry; exc = excited; fea = fearful; sur = surprised; fru = frustrated; hap = happy; neu = neutral; sad = sad; dis = disgusted.

The average values of emotional arousal level and the EALVI for each emotion category were compared. Figure 2 shows the number of utterances in each category. In addition, Fig. 3 shows the average values of emotional arousal level and the EALVI for each category. Here, the categories on the horizontal axis are arranged in descending order of average emotional arousal level. Except for the three categories of “fearful,” “surprised,” and “disgusted,” the order relationship between emotional arousal level and the EALVI was the same. As can be seen from the figure, both emotional arousal levels and the EALVI values tended to be high in the categories of “angry” and “excited,” and low in the categories of “neutral” and “sad,” which is consistent with our daily feelings. The number of utterances included in the three categories, in which the order of emotional arousal level and the EALVI values did not match, was extremely small, compared to other categories. The utterances (percentage) of the categories “fearful,” “surprised,” and “disgusted” were 40 (0.53%), 103 (1.42%), and 2 (0.03%), respectively.

### HAM-D score

There are various discussions on the classification of depression severity using the HAM-D score^25^. In this study, the dataset was divided into two groups, a “no depression” group with a HAM-D score of less than 8 and a “depression” group with a HAM-D score of 8 or more, using Hashim’s method^26^. Besides the HAM-D, the primary mental health assessment tools currently in use include self-administered questionnaires (e.g., the general health questionnaire^10^ and the Beck Depression Inventory^11^). However, self-administered questionnaires have reporting bias issues^27^. Reporting bias refers to participants’ intentional disclosure or suppression of certain information (e.g., medical history, smoking history). Hence, in this study, the HAM-D was used to assess depression severity.

Table 3 shows the mean HAM-D score of each group according to the facility. According to the Wilcoxon rank sum test, a significant difference was found between the HAM-D scores of each group, in both the GTC and the NDMC Hospital (*p* = 1.2** × **10^−8^, *p* = 1.6** × **10^−13^, respectively).

**Table 3:** Mean scores on the Hamilton Rating Scale for Depression (HAM-D).

Group	Sex	Number of participants	Mean age ± SD	Mean HAM-D Score ± SD	
**(a) GTC**					
No depression (HAM-D < 8)	Female	3	29.7 ± 8.1	5.3 ± 1.7	4.8 ± 1.3
	Male	7	29.6 ± 4.0	4.6 ± 1.6	
Depression (HAM-D ≧ 8)	Female	52	31.8 ± 8.5	23.9 ± 8.5	24.4 ± 8.5
	Male	26	33.3 ± 6.7	25.4 ± 8.4	
**(b) NDMC**					
No depression (HAM-D < 8)	Female	38	63.7 ± 12.8	1.8 ± 1.9	2.2 ± 2.2
	Male	27	52.3 ± 15.2	2.7 ± 2.4	
Depression (HAM-D ≧ 8)	Female	6	51.2 ± 7.6	9.3 ± 1.8	15.3 ± 7.2
	Male	19	43.8 ± 7.6	17.2 ± 7.2	

### The EALVI as an index of depression severity

**Figure 4:**
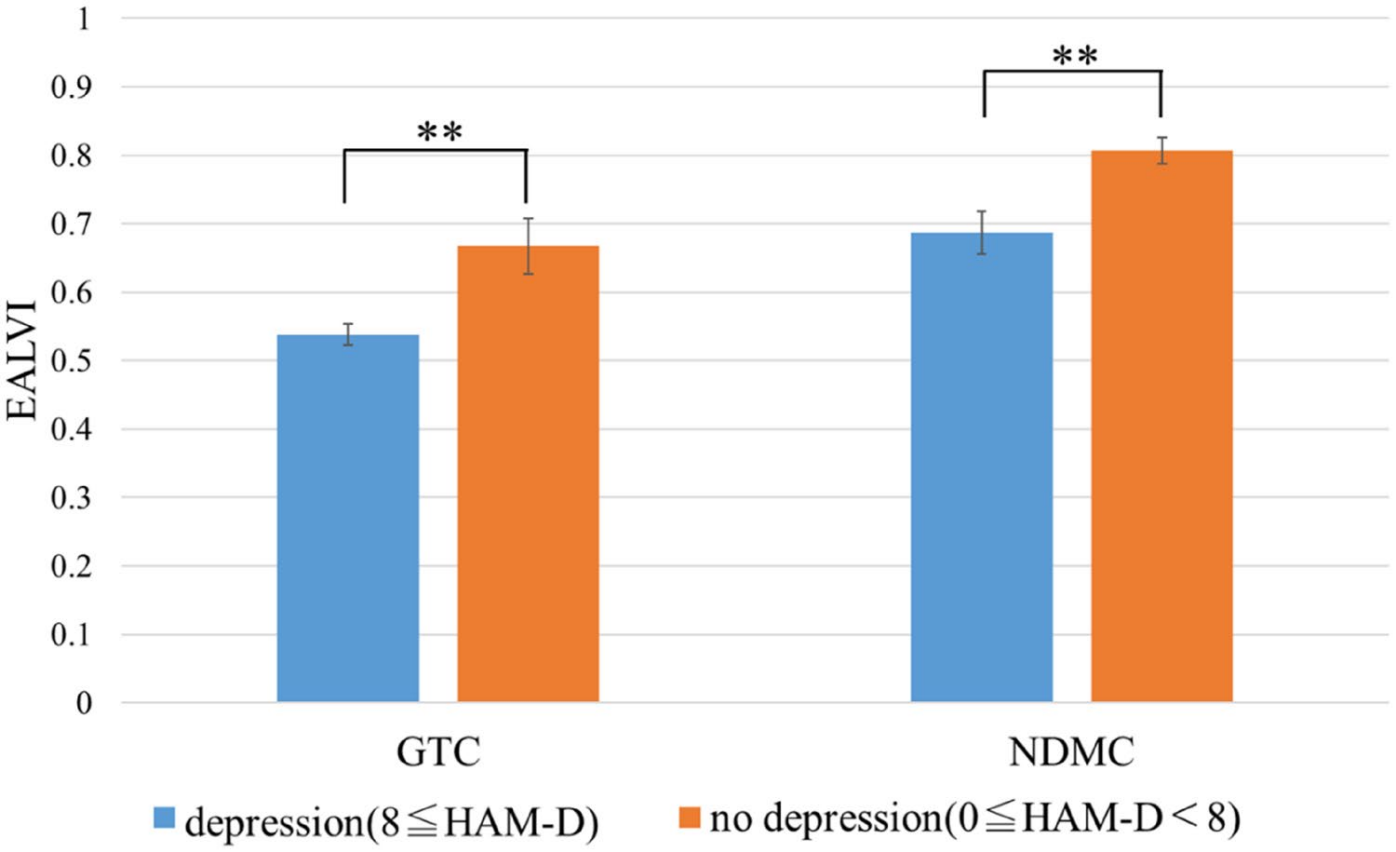
The mean EALVI value for the “depression” and “no depression” groups, by facility. *Note*: Error bars represent standard error. **(*p* < 0.01).

Data of patients with major depression were collected from two health care facilities. As shown in Table 2, the age groups of patients at both facilities were quite different. In addition, the sound field environments may be different in both facilities. Therefore, a separate analysis was performed for each hospital. Figure 4 shows the mean EALVI values of each group for each facility. However, the EALVI value of each participant was obtained by calculating the mean value of the EALVIs for the 10 phrases. At the GTC, the mean EALVI values of the “no depression” and “depression” groups were 0.67 ± 0.041 (n = 10) and 0.54 ± 0.015 (n = 78), respectively. At the NDMC Hospital, the mean EALVI values of the “no depression” and “depression” groups were 0.81 ± 0.020 (n = 65) and 0.69 ± 0.031 (n = 25), respectively.

The results of the Wilcoxon rank sum test showed a significant difference between the mean EALVI values of each group in both the GTC and the NDMC Hospital (*p* = 8.9** × **10^−3^, Cliff's delta = 0.51 and *p* = 1.6** × **10^−3^, Cliff's delta = 0.43, respectively). The AUC when the groups were identified using the EALVI was 0.76 for the GTC (cutoff point = 0.62, sensitivity = 0.80, specificity = 0.73) and 0.72 for the NDMC Hospital (cutoff point = 0.63, sensitivity = 0.89, specificity = 0.48). The correlation coefficient between the HAM-D score and the EALVI was − 0.33 (n = 88, *p* = 1.8** × **10^−3^) in the GTC and − 0.43 (n = 90, *p* = 2.2** × **10^−5^) in the NDMC Hospital.

Next, to determine the effect of gender and age, we performed multiple regression analyses with HAM-D score as the dependent variable and EALVI, age, and gender as the independent variables. We used a dummy variable for gender, setting 1 for males and 0 for females. Multicollinearity checks were carried out between the independent variables. The variance inflation factor was less than 2 among all variables and there was no collinearity. The results of the analyses are shown in Table 4. For both the GTC and NDMC, there was a negative contribution of the EALVI in predicting HAMD scores. There was no significant contribution from gender. There was a negative contribution of age only for the NDMC.

**Table 4:** Results of multiple regression analyses on HAM-D scores.

Independent variable	GTC		NDMC	
	*β*	*r*	*β*	*r*
EALVI	− 0.35**	− 0.33**	− 0.28*	− 0.43***
Age	0.0042	− 0.032	− 0.30**	− 0.43***
Sex	− 0.14	− 0.092	0.12	0.40***
R-square	0.13**		0.30***	
Adjusted R-square	0.097		0.27	

***(*p* < 1.0 × 10^−3^), **(*p* < 1.0 × 10^−2^), *(*p* < 5.0 × 10^−2^). *β* denotes the standardized partial regression coefficient. *r* represents the correlation coefficient between HAM-D score and each independent variable.

**Figure 5:**
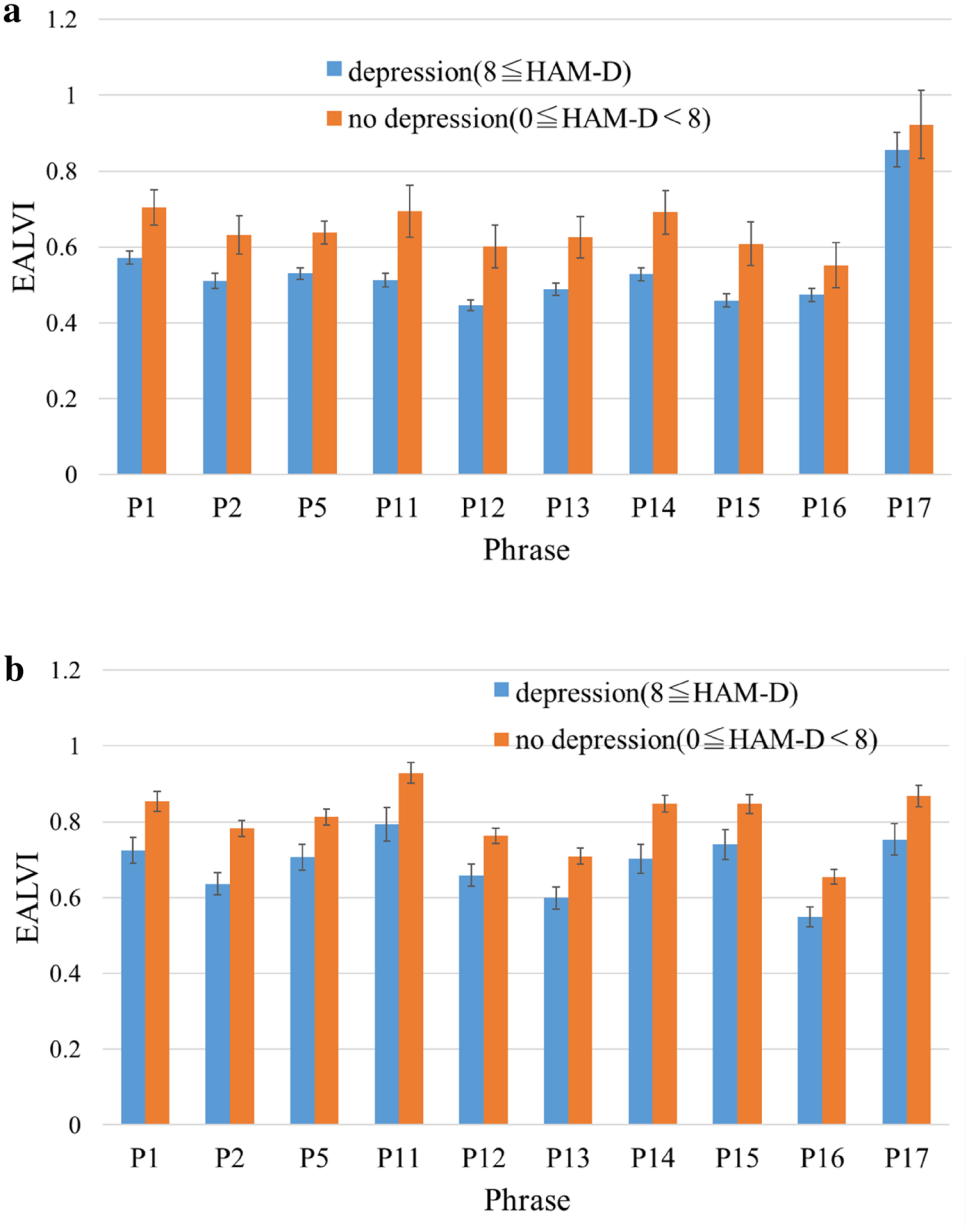
(**a**) The mean of the EALVI values of the “no depression” and “depression” groups for each phrase at the GTC. *Note*: Error bars represent standard error. (**b**) The mean of the EALVI values of the “no depression” and “depression” groups for each phrase at the NDMC Hospital. *Note*: Error bars represent standard error.

Next, we examined the effect of different phrases on the EALVI values obtained from each facility. Figure 5a,b show the mean EALVI values for each phrase in the “no depression” and “depression” groups. In all phrases, EALVI values of the “no depression” group were higher than those of the “depression group” at both facilities.

Table 5 shows a summary of classification performance between the “no depression” and “depression” groups by the EALVI. The AUC tended to be higher for the GTC. On the other hand, the correlation coefficient with the HAM-D tended to be higher for the NDMC Hospital.

**Table 5:** A summary of classification performance between no depression and depression groups by the EALVI.

Phrase	*p* value^a^		Effect size^b^		Area under the curve (AUC)		Correlation coefficient	
	GTC	NDMC	GTC	NDMC	GTC	NDMC	GTC	NDMC
P1	1.9** × **10^−2^*	5.6** × **10^−3^**	0.46	0.38	0.73	0.69	− 0.27*	− 0.34**
P2	2.6** × **10^−2^*	**1.5 × 10**^−4^***	0.43	**0.52**	0.72	**0.76**	− 0.28**	− 0.49***
P5	**7.3 × 10**^−3^**	8.9** × **10^−3^**	**0.52**	0.36	**0.76**	0.68	− 0.22*	− 0.38***
P11	8.6** × **10^−3^**	5.3** × **10^−3^**	0.51	0.38	0.76	0.69	− 0.25*	− 0.37***
P12	1.2** × **10^−2^*	5.8** × **10^−3^**	0.49	0.38	0.75	0.69	− 0.25*	− 0.34**
P13	1.7** × **10^−2^*	6.6** × **10^−3^**	0.46	0.37	0.73	0.69	− 0.30**	− 0.38***
P14	7.6** × **10^−3^**	1.9** × **10^−3^**	0.52	0.43	0.76	0.71	− 0.31**	− 0.41***
P15	1.9** × **10^−2^*	1.6** × **10^−2^*	0.46	0.33	0.73	0.67	− 0.26*	− 0.35***
P16	2.5** × **10^−1^	2.4** × **10^−3^**	0.22	0.41	0.61	0.71	− 0.21	− 0.39***
P17	3.6** × **10^−1^	5.0** × **10^−2^*	0.17	0.27	0.59	0.63	− 0.21*	− 0.35***
Total	8.9** × **10^−3^**	1.6** × **10^−3^**	0.51	0.43	0.76	0.72	− 0.33**	− 0.43***

The table shows the *p* values obtained by the Wilcoxon rank sum test, the effect sizes by Cliff’s delta, the AUC, and the correlation coefficient between the HAM-D and EALVI for each phrase of both facilities. The minimum p-value, maximum effect size, maximum AUC, and maximum correlation for each hospital are shown in bold fonts.

***(*p* < 1.0** × **10^−3^), **(*p* < 1.0** × **10^−2^), *(*p* < 5.0** × **10^−2^).

^a^By Wilcoxon rank sum test.

^b^By Cliff’s delta.

## Discussion

In this research, we proposed a new voice index of emotional arousal level, the EALVI, and investigated the relationship between the index and depression severity. The correlation coefficient between the EALVI and emotional arousal level evaluated by the annotators in IEMOCAP was 0.52. The correlation coefficients between the EALVI and HAM-D scores for depression severity were − 0.33 and − 0.43 at the GTC and NDMC, respectively.

As mentioned in the introduction, this study used the same data set used in Shinohara et al.^16^. When the EALVI was used to classify low emotional arousal voices with a level of 2 or less and high emotional arousal voices with a level of 4 or more, the AUC was 0.93. On the other hand, for the ALVI proposed in Shinohara et al.^16^, the AUC was 0.89. The AUC, at the moment when the “no depression” and the “depression” groups were identified using the EALVI, was 0.76 for GTC and 0.72 for NDMC. On the other hand, when ALVI was used for classification, the AUC was 0.66 for GTC and 0.70 for NDMC. Thus, the EALVI outperformed the ALVI in both emotional arousal and depression severity classification performance.

The EALVI, a voice index proposed by this paper, showed a significant correlation with the emotional arousal level evaluated by annotators. In the comparison of the EALVI and emotional arousal level by emotion category, the order relation between “fearful,” “surprised,” “frustrated,” and “happy” was reversed. However, as can be seen from Fig. 3, the values of the emotional arousal level are almost the same for these four emotions, and it can be said that it is difficult even for human beings to distinguish among them. Regarding the emotion category “disgusted,” the values of the emotional arousal level and EALVI were significantly different, but this may be because the sample size was too small (n = 2). In the IEMOCAP database, the inter-rater Cronbach’s alpha for emotional arousal level was not high at 0.607. Therefore, caution should be exercised in assessing the correlation between emotional arousal level and the EALVI.

With regard to depression severity, there was also a significant correlation between the HAM-D score and the EALVI value. The AUC was over 0.7 in both facilities. MFCC2 showed a very high AUC of 0.88 in discriminating healthy individuals from patients suffering from MDD^15^. However, there was no significant correlation between the MFCC2 and the severity of depression. On the other hand, the EALVI value significantly correlated with the HAM-D score. It should be noted that the AUC of the EALVI was lower than that of MFCC2, but in this study, all the participants were outpatients suffering from depression. In future studies, we would like to compare the results of healthy individuals with those of patients suffering from depression.

The model classifying a depressive state versus a euthymic state had an AUC of 0.78 in the study by Faurholt-Jepsen et al.^28^. Here, a depressive state is defined as one with a HAM-D score ≥ 13 and a Young Mania Rating Scale (YMRS) score < 13, while a euthymic state is defined as one with a HAM-D score < 13 and a YMRS score < 13. Although no simple comparison is possible, the AUC shown here was about the same value as that of the EALVI. However, it may be advantageous because the EALVI is unlikely to be overfitted as it consists of only one feature.

As shown above, the EALVI was shown to be associated with both emotional arousal level and depression severity. However, this study did not directly show a relationship between emotional arousal level and depression severity. In the future, we would like to directly examine the relationship between the EALVI, emotional arousal level, and depression severity using the same dataset. Furthermore, it should be noted that the emotional arousal level was an evaluation value given by the annotator and did not reflect the participant’s own evaluation. In the future, it will be necessary to investigate the relationship between the EALVI and physiological indicators.

In addition, we need to clarify the qualitative meaning of the EALVI. It is necessary to clarify how we perceive speech that has a high acceleration toward the center.

This study has some limitations. First, all participants had to use the same phrases for an accurate evaluation. Our future task would be to analyze the EALVI of spontaneous speech. Second, the sample size of the group was small. We collected voices from two health care facilities, the GTC and the NDMC Hospital, but the age group and the distribution of HAM-D scores were quite different at both facilities.

The mean age of the participants was higher in the NDMC Hospital. Conversely, the HAM-D score was higher in the GTC. The reason for this may be that the GTC is located in central Tokyo and many outpatients are young office workers who commute to the city center; while the NDMC Hospital is in the suburbs and several patients are elderly people living in the locality. The NDMC Hospital is a university hospital and many patients have already been treated at other hospitals. Future studies should include a larger sample size acquired in the similar environments. Third, the present study does not address the issue of specificity. Since there are various diseases other than depression that are related to emotional arousal level, low emotional arousal does not necessarily mean high depression severity. For example, apathy is common in many different neurodegenerative illnesses including Alzheimer’s disease (AD), frontotemporal dementia (FTD), Parkinson’s disease, and dementia with Lewy bodies, and overlaps with other neuropsychiatric syndromes such as depression. In addition, impairment of the same brain networks involved in arousal, threat response, and reward processing is associated with apathy in AD and FTD^29^. In the future, we need to develop a new index that can differentiate between MDD and the aforementioned diseases.

## Data Availability

According to Japanese law, the sensitivity of audio files is similar to that of any other personal information and cannot be published without consent. In this research protocol, we did not obtain consent from the participants to publish the raw audio files as a corpus. The datasets used and/or analyzed during the current study are available from the corresponding author upon reasonable request.
